# Lymphangitis Treated by Zinc Ionization

**Published:** 1912-10

**Authors:** 


LYMPHANGITIS TREATED BY ZINC IONIZATION. Marques, *Arch. d'Elect., Med.,* Dec. 10, 1911. The lymphangitis occurred three days after a punctured wound of the fore-arm. 2% solution of zinc chlorid was applied on absorbent cotton, connected to the positive pole and 40 milliampers were passed for 30 minutes next day, the process seemed to be at an end but there was a recrudescence after three days, permanent recovery following a second treatment.

